# Altered insular functional connectivity correlates to impaired vigilant attention after sleep deprivation: A resting-state functional magnetic resonance imaging study

**DOI:** 10.3389/fnins.2022.889009

**Published:** 2022-07-26

**Authors:** Weiwei Fu, Cimin Dai, Jie Chen, Letong Wang, Tao Song, Ziyi Peng, Mengmeng Xu, Lin Xu, Yuguo Tang, Yongcong Shao

**Affiliations:** ^1^School of Biomedical Engineering (Suzhou), Division of Life Sciences and Medicine, University of Science and Technology of China, Hefei, China; ^2^Suzhou Institute of Biomedical Engineering and Technology, Chinese Academy of Sciences, Suzhou, China; ^3^School of Psychology, Beijing Sport University, Beijing, China

**Keywords:** sleep deprivation, psychomotor vigilance task, insula, functional connectivity, resting-state functional magnetic resonance imaging

## Abstract

**Objectives:**

This study used resting-state functional magnetic resonance imaging (rs-fMRI) scans to assess the dominant effects of 36 h total sleep deprivation (TSD) on vigilant attention and changes in the resting-state network.

**Materials and methods:**

Twenty-two healthy college students were enrolled in this study. Participants underwent two rs-fMRI scans, once in rested wakefulness (RW) and once after 36 h of TSD. We used psychomotor vigilance tasks (PVT) to measure vigilant attention. The region-of-interest to region-of-interest correlation was employed to analyze the relationship within the salience network (SN) and between other networks after 36 h of TSD. Furthermore, Pearson’s correlation analysis investigated the relationship between altered insular functional connectivity and PVT performance.

**Results:**

After 36 h of TSD, participants showed significantly decreased vigilant attention. Additionally, TSD induced decreased functional connectivity between the visual and parietal regions, whereas, a significant increase was observed between the anterior cingulate cortex and insula. Moreover, changes in functional connectivity in the anterior cingulate cortex and insula showed a significant positive correlation with the response time to PVT.

**Conclusion:**

Our results suggest that 36 h of TSD impaired vigilant visual attention, resulting in slower reaction times. The decrease in visual-parietal functional connectivity may be related to the decrease in the reception of information in the brain. Enhanced functional connectivity of the anterior cingulate cortex with the insula revealed that the brain network compensation occurs mainly in executive function.

## Introduction

Sleep is an innate biological rhythm that is a physiological need to maintain daily life (Lester et al., 1983). Most of us spend approximately one-third of our lives asleep. Adequate sleep restores the brain and body, thus replenishing depleted bodily functions (Abbott et al., 2018). Sleep deprivation (SD) is a state caused by the interaction of the internal state and external environment. It is operationally defined as the state of an individual who does not sleep for more than 4 h in total, during a 24 h day (Jovanović, 1991). However, with the rapid pace of modern life, high stress, and frequent use of electronic network products, sleep deprivation has become a common phenomenon (Barnes et al., 2011). In the “White Paper on Exercise and Sleep 2021,” the big data shows that “the incidence of insomnia among Chinese adults is as high as 38.2%, and there are over 300 million people with sleep disorders in China today,” especially in young people (Patrick et al., 2017). Studies have shown that more than 70% of young people sleep less than 7 h a day, and at least 60% suffer from severe sleep deprivation (Owens et al., 2017). Simultaneously, people must give up sleep and stay awake all night because of the demands of their work (Bin et al., 2012). Workers on manufacturing lines, doctors on night shifts, truck drivers on long hauls, and firefighters are often sleep-deprived. This leads to a dramatic decrease in attention, prolonged reaction times, and a significant decrease in judgment and thinking skills (Hyams et al., 2010). A series of high loads and shift systems of continuous working conditions will cause irreversible damage to the human body and mind and are prone to many serious safety accidents (Patanaik et al., 2015). Sleep deprivation negatively affects tens of millions of lives, their education, employment, and mental health.

Vigilant attention is a special type of attention in which individuals maintain a state of constant awareness and vigilance to stimuli of adaptive importance that may appear, but have not yet appeared (Hudson et al., 2020). The attentional network model, proposed by Posner and Petersen (1990) divides attention into three interconnected subnetworks: a pre-attentive network, a post-attentive network, and a vigilance system, corresponding to the three main functions of executive control, directed inhibition, and vigilance of attention. The psychomotor vigilance task (PVT) paradigm, which is highly reproducible, has small learning effects, is reliable, has become the gold standard for assessing the effects of sleep deprivation on vigilance, and is an ideal tool for behavioral assessment (Van Dongen et al., 2003). As the duration of the waking state increases, the individual’s vigilance function continues to decline, making it difficult to maintain focused attention for long periods. Therefore, the response time of vigilance tasks is particularly sensitive to sleep deprivation (Lim and Dinges, 2010). It leads to prolonged PVT reaction time (Doran et al., 2001); in addition, the impairment of vigilance by sleep deprivation is reflected in the reaction time, and response lapse is also a sensitivity indicator. Some researchers have found that individual response times are significantly longer, and response lapse is significantly increased after sleep deprivation (Teng et al., 2019), suggesting that sleep deprivation leads to physical and mental fatigue in individuals, which leads to slower responses and increased errors. Zepf et al. (2019) found that adults with ADHD have abnormal anterior insular connections and that abnormal connection is associated with poor attention, difficulty in social functioning, and impaired cognitive control. Their research found the brain network mechanism of ADHD in the resting state. However, the attention level of ADHD is already comparatively lower. Tu et al. (2020) revealed that frontal functional disconnection may underlie the pathogenesis responsible for vigilance attention. However, the pattern of attention brain networks in healthy people after 36 h of sleep deprivation that have few understandings. The effects of sleep deprivation on brain function are temporary and reversible, through a period of recovery sleep, previously disturbed brain function is restored to balance. Therefore, we focused the changes between attention performance and the intra-network and inter-network functional connectivity of healthy adults from resting state perspectives after 36 h of SD. We can use SD to elucidate the underlying mechanisms of vigilance attention which is an important goal in basic and clinical neuroscience.

Sleep deprivation severely impairs an individual’s cognitive functions, such as sensory perception (Aguiar and Barela, 2015), visual information processing (Cohen-Zion et al., 2016), emotional regulation (Killgore et al., 2008), working memory (Gerhardsson et al., 2019), vigilant attention (Roca et al., 2012), and executive control functions (Corsi-Cabrera et al., 2015). The most severe impairment is decreased visual vigilance, which is considered the basis for other cognitive impairments (Lim and Dinges, 2010). Event Related Potential provides electrophysiological evidence that sleep deprivation impairs vigilance in the temporal dimension. One study found a decrease in wave amplitude and prolongation of latency in individuals with longer sleep deprivation, such as N1, N382, P300, and P718, suggesting that sleep deprivation slows down the detection of visual stimuli, decreases the correct rate, and diminishes the level of vigilance (Humphrey et al., 1994; Corsi-Cabrera et al., 1999). Other researchers have investigated vigilance using high-resolution spatial brain-imaging techniques. After sleep deprivation, visual vigilance function is not only associated with decreased prefrontal function, but also with decreased parietal and visual cortices, as well as thalamic function (Chee et al., 2011), significantly slower processing of visual information, and reduced executive control functions.

Resting-state functional magnetic resonance imaging (rs-fMRI) is a neuroimaging technique used to study the functional connectivity of the resting brain networks, by comparing the time-domain correlation of signal fluctuations in blood oxygen levels between different brain regions, to determine the functional connectivity of each brain region (Greicius et al., 2003). The human cognitive system is composed of eight main brain networks: default mode network (DMN), sensorimotor network (SMN), visual network (VN), salience network (SN), dorsal attention network (DAN), ventral attention network (VAN), frontal-parietal control network (FPN), and subcortical network (Yeo et al., 2011). It has been found that sleep deprivation impairs cognitive processes, but functional compensation occurs. Recent studies have found that DMN is not simply one homogeneous network but at least two functionally distinct sub-networks. One sub-network consists of the dorsal and anterior regions, called the dorsal DMN, and is involved in introspective, self-oriented processes; the other consists of posterior and medial temporal regions, called the ventral DMN, and is engaged in decision-making, which is a more complex process requiring higher cognitive function (Andrews-Hanna et al., 2010; Doucet et al., 2011; Chen et al., 2017). Functional connectivity within the dorsal DMN was reduced by SD, whereas enhanced functional connectivity was found within the ventral DMN as well as between the two sub-networks. Enhanced communication across two sub-networks correlated positively with working memory and negatively with PVT response time, suggesting compensatory functional connectivity between the dorsal and ventral DMN (Chen et al., 2018). The SN mediates the transition between the default mode network and frontoparietal control networks, to guide rational responses to salient stimuli (Menon and Uddin, 2010). The anterior insular cortex plays a central role in the SN. Sleep deprivation reduces the activity of the SN in attention tasks (Ma et al., 2015). It also reduces function in the right anterior insula which causes the frontoparietal control network activity for task execution, to switch back and forth with task-independent default network activity (Sridharan et al., 2008). This is most likely the cause of the unstable attentional state of the brain and the sharp decrease in vigilance function after sleep deprivation.

Most studies have found that sleep deprivation can cause severe impairment in visual vigilance function and have provided sufficient evidence of the brain regions’ electrophysiological and functional activation. However, few studies have investigated the dominant effects of 36 h total sleep deprivation (TSD) on vigilant attention in individuals from the perspective of functional connectivity in resting-state brain networks. Therefore, this study hypothesized that individuals with sleep deprivation would have severely impaired vigilance function, with significantly longer reaction times, increased response lapse, and decreased functional connectivity with the anterior insula, the core node of the SN for attentional information processing. This study aimed to establish a theoretical basis for the decline of vigilance function and intervention in individuals following sleep deprivation, by investigating the impact of sleep deprivation on neural networks and changes in functional connectivity of brain networks.

## Materials and methods

### Participants

Twenty-two healthy male college students (mean age: 24 years; age range 21–28 years), volunteered to participate in this experiment. The participants were all from Beijing, were right-handed, had normal or corrected visual acuity, had not previously participated in psychological or physical-related tests, had undergone a rigorous physical examination by a psychiatrist to exclude psychiatric and somatic disorders, and had no tobacco or alcohol dependence or other undesirable habits. Participants’ intelligence quotient (IQ) scores on the Raven’s Standard Progressive Matrices were above the population average (IQ > 110). Participants’ scores on the Pittsburgh Sleep Quality Index (PSQI < 5) indicated good sleep quality. The experimental procedure and precautions to be taken during the experiment were explained in detail to them before the experiment. All participants completed an informed consent form and received the appropriate participant fee at the end of the experiment. The Ethics Committee of the Beihang University (Beijing, China) approved the experiment.

### Experimental materials

#### Psychomotor vigilance task

**FIGURE 1:**
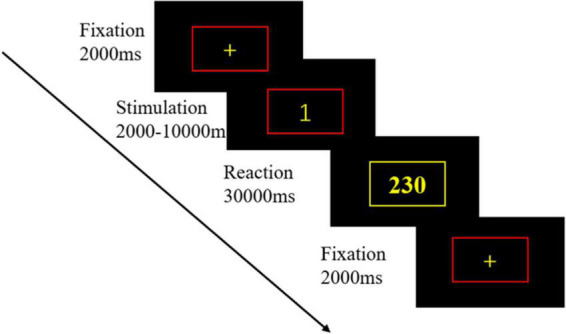
The psychomotor vigilance task (PVT) paradigm. Firstly, a yellow fixation cross with a red box appeared in the center of a black background on the screen and remained for 2 s. Then, a yellow numerical stimulus was presented in the red box for a random duration of 2–10 s. After that, participant had to respond to the mouse button press as soon as possible. After pressing the button, the box turned yellow, and the number in the box was the participant’s response time.

The PVT was used to test the vigilance status of the participants. PVT is one of the most used experimental paradigms in sleep deprivation research and is sensitive to sleep deprivation in several metrics, including mean reaction time, median reaction time, 10% fastest reaction time, 10% slowest reaction time, and response lapse. The experimental procedure used in this study was based on the E-prime 3.0 platform, with a duration consistent with the prevailing standard version, set at 10 min (Lim and Dinges, 2010), and the response device used was a computer mouse. First, a yellow fixation cross with a red box appeared on the screen in the center of a black background and remained there for 2 s. Second, a yellow numerical stimulus was presented inside the red box for a random duration of 2–10 s. After that, participants had to respond by clicking the mouse as rapidly as possible. After clicking the mouse, the box turned yellow and the number in the box was the participant’s response time. After few moments, the box turned red again, and the next trial began. Figure 1 illustrates the PVT paradigm.

#### Resting-state magnetic resonance imaging scans

All participants underwent two rs-fMRI scans, once in rested wakefulness (RW) and once after 36 h of TSD. Following the rs-fMRI scans, a PVT was conducted to measure the vigilant attention of participants in both RW and TSD states (Zhang et al., 2021). Behavioral data and resting-state MRI were collected twice. Participants were asked to keep their eyes open during the scan and not think about anything else. At the end of the scan, participants were asked if they had fallen asleep. The entire scan lasted for approximately 7 min. Brain imaging data of the participants were stored and processed for subsequent analysis of brain network characteristics.

### Experimental procedure

**FIGURE 2:**
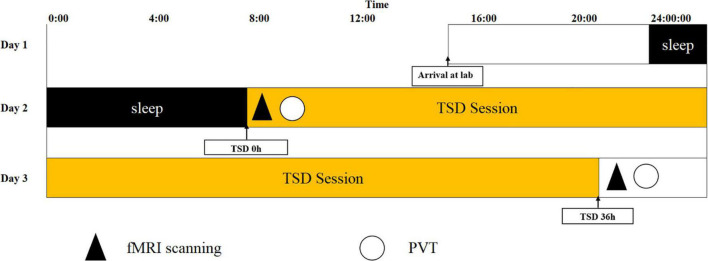
Experimental flow chart. Total sleep deprivation (TSD) began after a routine nocturnal sleep period, at 08:00 on Day 2, and ended at 20:00 at Day 3. Participants were required to stay awake for 36 h during the whole TSD session. Participants performed psychomotor vigilance tasks (PVT) and functional magnetic resonance imaging (fMRI) scanning at 20:00 on Day 2. After 36 h TSD, fMRI scanning, and PVT was performed at approximately 20:00 on Day 3 for all participants.

The experiment used a repeated measures design. Participants were part of a single 36 h of TSD experiment. At 16:00 on the day before the experiment, the participants arrived at the laboratory, were given details about the experiment as well as precautions. They completed a general informed consent form to sleep in the laboratory that night, guaranteeing the participant 8 h of sleep. Participants began sleep deprivation at 8:00 a.m. on Day 2, with the 1st MRI scan set as a baseline measurement and a PVT was conducted following the MRI scan. After completing sleep deprivation for 36 h at 8 p.m. on Day 3, an MRI scan was performed, and thereafter, the second PVT was conducted. Two participants entered the experiment simultaneously, ensuring that three people were in the laboratory. This comprised the health-care worker and the two participants. The participants were supervised to ensure that they were prevented from sleeping (including napping) throughout the sleep deprivation period. Behavioral metrics were recorded, including the participants’ reaction time to task completion, response lapse, median response, 10% fastest response time, and 10% slowest response time. The functional connectivity imaging technique selected 26 regions of interest (ROIs) from six brain networks: the DMN, SMN, VN, SN, DAN, and FPN. The SN was used as the seed from which to explore the functional connectivity between and within the SN and each brain network. Magnetic resonance scans were performed in the magnetic resonance room of Beijing Military General Hospital. Before experiment onset, the participants prepared themselves (removing metal objects, wearing shoe covers, earplugs, etc.), and then laid flat on the MRI scanner with a sponge block padded on the head, to reduce their head movement during the scan. The scans were performed with a GE 3.0 T Discovery 750 MRI system (General Electric Medical System, Milwaukee, WI, United States), using an eight-channel head coil to acquire MRI signals. The participants were asked to keep their eyes open, remain relaxed, and keep their heads as still as possible during the entire scan. A camera was installed in the cavity of the MRI scanner to monitor whether the participant’s eyes remained open in real-time, to ensure that they did not fall asleep during the entire MRI scan. Using an echo-planar imaging sequence with 210 frames, resting-state scans were performed. The acquisition parameters were as follows: repetition time = 2,000 ms, echo time = 30 ms, field of view = 210 mm × 240 mm, slice thickness = 3 mm, slice gap = 1 mm, flip angle = 90°, acquisition matrix (matrix) = 64 × 64, 35 oblique slices (parallel to the AC-PC line), and volume = 210. The high-resolution T1 structure image was acquired using the FSPGR-BRAVO sequence, with the following acquisition parameters: repetition time = 8,208 ms, inversion time = 450 ms, echo time = 3.22 ms, flip angle = 12°, field of view = 240 × 240 mm, voxel size = 1 mm × 1 mm × 1 mm, and slice number = 192. The experimental flowchart is shown in Figure 2.

### Data analysis

#### Behavioral data analysis

Psychomotor vigilance task data were received from 23 participants, of which one participant was excluded owing to invalid data. Therefore, the final behavioral data of the 22 participants were analyzed. The behavioral data measures were mean reaction time, response lapse, median reaction time, 10% fastest reaction time, and 10% slowest reaction time. The mean reaction time is the average of the participants’ reaction times for each trial of the PVT. We defined reaction times ≤100 (ms) as incorrect responses with a total of two occurrences, and reaction times ≥500 (ms) as reaction lapse. The median reaction time was the middle reaction time of all trials in descending order of reaction time, 10% of the fastest RT, 10% of the slowest reaction time-averaged over the first 10%, and last 10% of all trials. Paired samples *t*-tests were performed using SPSS ver28.0 (IBM, Armonk, NY, United States), to compare the differences in reaction time, response lapse, median reaction time, 10% fastest reaction time, and 10% slowest reaction time, before and after sleep deprivation.

#### Functional magnetic resonance imaging data processing

**FIGURE 3:**
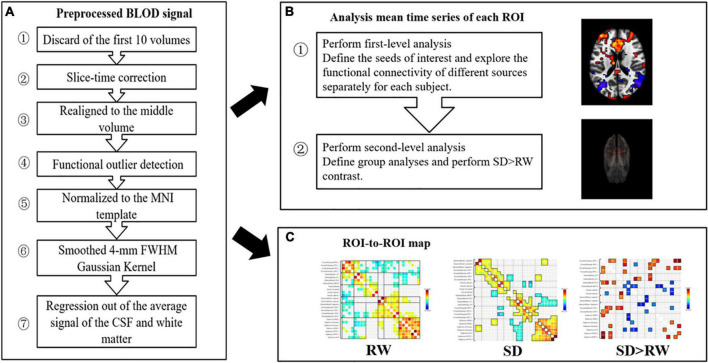
Preprocessing and analysis pipeline. **(A)** Preprocessing pipeline; **(B)** analysis pipeline: first and second level analyses; **(C)** ROI-to-ROI map: 26 regions of interest (ROis) were selected from the six large scale networks: default mode network (DMN), sensorimotor network (SMN), visual network (VN), salience network (SN), dorsal attention network (DAN), ventral attention and frontal-parietal control network (FPN), SN as the seed, ROI-to-ROI analysis. SD, sleep deprivation; RW, rested wakefulness; ROI, regions of interest.

**FIGURE 4:**
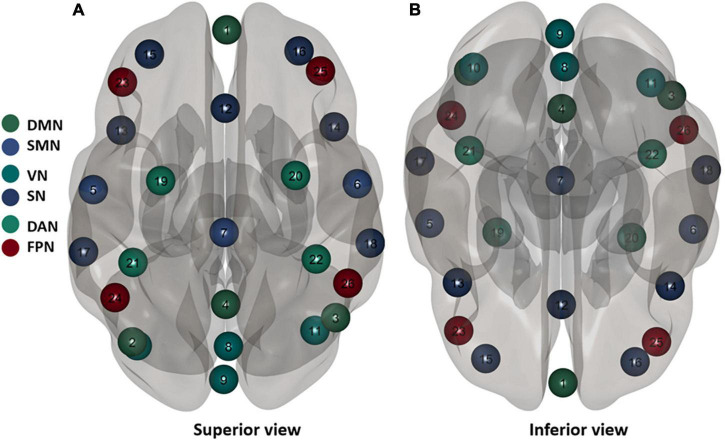
Twenty six brain regions of interest (ROis) distribution in the brain. Default mode network (DMN), sensorimotor network (SMN), visual network (VN), salience network (SN), dorsal attention network (DAN), ventral attention, and frontal-parietal control network (FPN). These numbers correspond to the number of brain regions in Table 1.

The resting-state fMRI data were based on the MATLAB (MathWorks, Inc., Natick, MA, United States) platform and the functional connectivity tool CONN 18b (Neuroimaging Informatics Tools and Resources Clearinghouse), for the process includes pre-processing, and functional connectivity analysis. First, we scrubbed the data, and the time point was set to 10; that is, the first 10 repetition-time images were scrubbed to avoid the effect of magnetic field instability at the beginning of the scan and to ensure the integrity of the data. Next, slice-time correction was performed to eliminate the variability of scan times between different slices, thus correcting the acquisition times of all slices to the same time point. The remaining rs-fMRI data were realigned to the middle volume of each session using the SPM12 realign and unwarp procedure to perform the head motion correction. Head motion correction was used to eliminate participants with excessive head motion parameters to facilitate subsequent statistical analysis; thereafter, functional outlier detection was used to remove extreme values, and then spatial normalization was performed, using the currently widely used template of the Montreal Neurological Institute (MNI). The standard spatial template established by the MNI, widely used today, was used to facilitate group-level analysis of all participants in a uniform space, and spatial smoothing was set to 4 mm × 4 mm × 4 mm to improve the effectiveness of statistical analysis. Before further processing, the quality of the pre-processed images was evaluated to prevent false-positive results. The quality report that was generated, built into the CONN, provided a preview of the participants’ image spatial normalization results. The alignment of the 22 resting-state images with the MNI standard space was as expected. The effects of some covariates were regressed using the CompCor function to reduce physiological noise factors and improve the signal-to-noise ratio. The preprocessing pipeline is illustrated in Figure 3A. According to the CONN template, 26 ROIs (Tzourio-Mazoyer et al., 2002) were selected from the six large-scale networks: DMN, SMN, VN, SN, DAN, and FPN (Yeo et al., 2011). The default mode network includes the medial prefrontal cortex (MPFC), left and right paracentral lobules [LP(L) and LP(R)], and posterior cingulate cortex (PCC); SMN includes the left and right lateral and superior; VN includes the medial, occipital, and left and right lateral; the SN includes the anterior cingulate cortex (ACC), left and right anterior insula, left and right rostrolateral prefrontal cortex [RPFC(L) and RPFC(R)], and left and right supramarginal gyrus [SMG(L) and SMG(R)]; the DAN includes left and right frontal eye field [FEF(L) and FEF(R)], and left and right intraparietal sulcus [IPS(L) and IPS(R)]; and the FPN includes the left and right lateral prefrontal cortex [LPFC(L) and LPFC(R)], and left and right posterior parietal cortex [PCC(L) and PCC(R)]. Table 1 lists the abbreviated names and coordinates of these 26 ROIs. The specific location distributions are shown in Figure 4. Twenty-six brain ROIs were selected to separately assess each participant’s functional connectivity from diverse sources in a first-order analysis. Then, the mean time course of each ROI was extracted by averaging the time series of all voxels in the corresponding ROI. ROI-ROI maps were generated by computing pairwise the Pearson’s correlation of the 26 ROIs. Thereafter, we chose seven ROIs of the SN as the seed for comparing the intra-and inter-networks of functional connectivity. The analysis pipeline is illustrated in Figure 3B. Data processing methods included general linear models fused with typical hemodynamic response functions. In the second-order analysis, comparisons between participants were made [SD > RW (1, −1)], based on a general linear model with random effects. The network-level correction was applied for multiple comparisons (false discovery rate, *p* < 0.05) (Whitfield-Gabrieli and Nieto-Castanon, 2012). The ROI-ROI map is illustrated in Figure 3C.

**TABLE 1:** Abbreviated names and coordinates of 26 regions of interests (ROIs) in the resting-state network.

Network	ROI	ROI name	Number	MNI center		
DMN	MPFC	Medial prefrontal cortex	1	1	55	−3
	LP(L)	Left paracentral lobule	2	−39	−77	33
	LP(R)	Right paracentral lobule	3	47	−67	29
	PCC	Posterior cingulate cortex	4	1	−61	38
SMN	Lateral(L)	Left Lateral	5	−55	−12	29
	Lateral(R)	Right Lateral	6	56	−10	29
	Superior	Superior	7	0	−31	67
VN	Mdial	Mdial	8	2	−79	12a
	Occipital	Occipital	9	0	−93	−4
	Lateral(L)	Left Lateral	10	−37	−79	10
	Lateral(R)	Right Lateral	11	38	−72	13
SN	ACC	Anterior cingulate cortex	12	0	22	35
	Alnsula(L)	Left anterior insula	13	−44	13	1
	Alnsula(R)	Right anterior insula	14	47	14	0
	RPFC(L)	Left rostrolateral prefrontal cortex	15	−32	45	27
	RPFC(R)	Right rostrolateral prefrontal cortex	16	32	46	27
	SMG(L)	Left supramarginal gyrus	17	−60	−39	61
	SMG(R)	Right supramarginal gyrus	18	62	−35	32
DAN	FEF(L)	Left frontal eye field	19	−27	−9	64
	FEF(R)	Right frontal eye field	20	30	−6	64
	IPS(L)	Left intraparietal sulcus	21	−39	−43	52
	IPS(R)	Right intraparietal sulcus	22	39	−42	54
FPN	LPFC(L)	Left lateral prefrontal cortex	23	−43	44	28
	PPC(L)	Left posterior parietal cortex	24	−46	−58	49
	LPFC(R)	Right lateral prefrontal cortex	25	41	38	30
	PPC(R)	Right posterior parietal cortex	26	52	−52	45

DMN, default mode network; SMN, sensorimotor network; VN, visual network; SN, salient network; DAN, dorsal attention network; FPN, frontoparietal control network; ROIs, regions of interest.

#### Statistical treatment

SPSS.28.0 was used to calculate the mean and standard deviation of the reaction time, response lapse, median reaction time, 10% fastest reaction time, and 10% slowest reaction time; paired samples *t*-tests were used to compare the differences between RW and TSD after 36 h. Finally, the behavioral data were correlated with the fMRI data by Pearson correlation, to explore their relationship.

## Results

### Behavioral results

For 22 participants with correct responses on the PVT, the mean reaction time, response lapse, median reaction time, 10% fastest reaction time, and 10% slowest reaction time were calculated and statistically analyzed, as shown in Table 2. Compared with RW, the mean reaction time was significantly slower after 36 h of TSD (*t*_21_ = 4.33, *p* = 0.006), the number of response lapse was significantly higher (*t*_21_ = 4.33, *p* < 0.001), the median reaction time was significantly longer (*t*_21_ = 3.27, *p* = 0.004), the 10% fastest reaction time was significantly longer (*t*_21_ = 3.50, *p* = 0.002), and 10% of the slowest responses were significantly prolonged (*t*_21_ = 2.71, *p* = 0.013).

**TABLE 2:** Behavioral change after sleep deprivation (SD).

Behavioral metrics	RW	SD	*t*	*P*
Number of lapses	2.14 ± 1.91	5.14 ± 4.38	4.33	<0.001
Reaction time (ms)	370.84 ± 57.02	442.18 ± 142.53	3.06	0.006
Median RT (ms)	345.55 ± 41.45	380.41 ± 57.72	3.27	0.004
Fastest 10% RT (ms)	281.45 ± 25.99	301.46 ± 28.33	3.50	0.002
Lowest 10% RT (ms)	586.30 ± 217.95	886.02 ± 674.43	2.71	0.013

Data are presented as mean ± standard deviation. RW, rested wakefulness; SD, sleep deprivation; RT, reaction time.

### Brain imaging results

#### Changes in functional connectivity of brain regions

**FIGURE 5:**
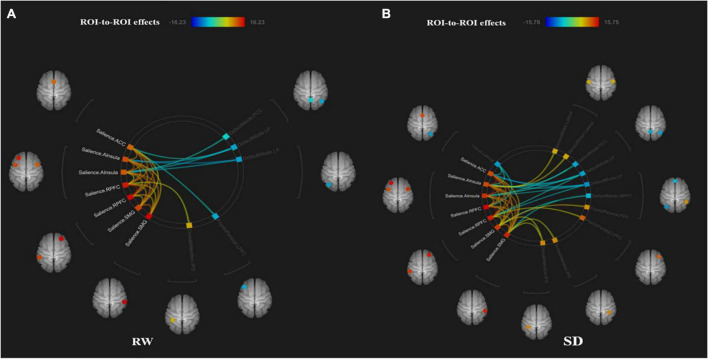
ROI-to-ROI connectivity of six networks in panels **(A)** rested wakefulness (RW) and **(B)** total sleep deprivation (TSD) scans [*p* < 0.001 FDR set wise corrected for all comparisons across the entire network]. ROI, region of interest; SD, sleep deprivation; DMN, default mode network; SMN, sensorimotor network; VN, visual network; SN, salience network; DAN, dorsal attention network; ventral attention and frontal-parietal control network (FPN).

**FIGURE 6:**
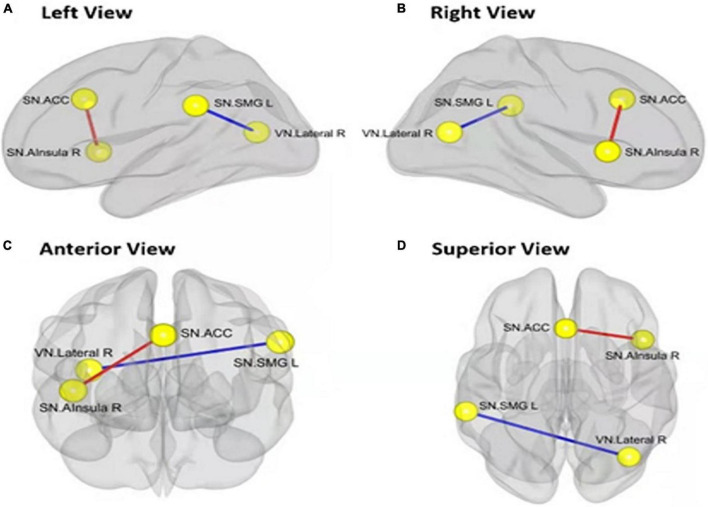
Alerted ROI-to-ROI functional connectivity of networks contrasting in rested wakefulness (RW) vs. total sleep deprivation (TSD) scans: Comparing to RW scan, functional connectivity of ACC-Alsula (R) [within salience network (SN)] is higher (the red line), and functional connectivity of SMG(L)-Lateral(R) (SN-VN) is lower (the blue line). All the results are shown at panels **(A)** left view, **(B)** right view, **(C)** anterior view, **(D)** superior view. (FDR set wise corrected for all comparisons across the entire network). ROI, region of interest; ACC, anterior cingulate cortex; SMG(L), left supramarginal gyrus; VN, visual network. *p < 0.05, **p < 0.01, ***p < 0.001.

The functional connectivity within the SN and between other networks, such as DMN, SMN, VN, DAN, and FPN, in RW and TSD is shown in Figure 5. The results showed that compared with RW, the functional connectivity between the left supramarginal gyrus [SMG(L)] and the right visual area (*t*_21_ = −3.68, *p-FDR* = 0.0349) was significantly decreased after SD. Within the SN, the functional connectivity between the anterior cingulate gyrus and the right insula (*t*_21_ = 3.55, *p-FDR* = 0.0472) was significantly enhanced. The results are shown in Figure 6 and Table 3.

**TABLE 3:** ROI-to-ROI functional connectivity statistics of a network: comparison of rested wakefulness (RW) and total sleep deprivation (TSD) scans (*t*-test).

Network	ROI-to-ROI	*t*	*p-unc*	*p-FDR*
SN-VN	SMG(L)-Lateral(R)	−3.68	0.0014	0.0349
Within-SN	ACC-Alnsula(R)	3.55	0.0019	0.0472

ROI, region of interest; FDR, false discovery rate; RW, rested wakefulness; TSD, total sleep deprivation. *p* < 0.05 FDR set wise corrected for all comparisons across the entire network. SN, salience network; VN, visual network; SMG(L), left supramarginal gyrus; ACC, anterior cingulate cortex.

#### Brain functional connectivity changes associated with psychomotor vigilance tasks

**FIGURE 7:**
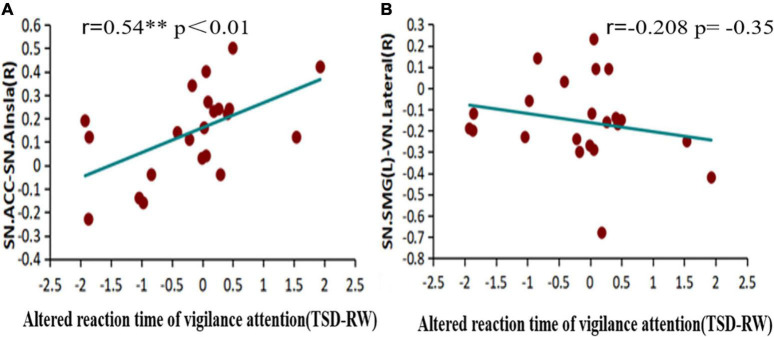
Alerted functional connectivity correlate to altered reaction time of vigilance attention: altered reaction time of vigilance attention is **(A)** positively correlated with the alerted functional connectivity of ACC-Alsula (R) [within salience network (SN)], and **(B)** negatively correlated with the alerted functional connectivity of SMG(L)-Lateral(R) (SN-VN) TSD, total sleep deprivation; RW, rested wakefulness; ACC, anterior cingulate cortex; SMG(L), left supramarginal gyrus; VN, visual network. *p < 0.05, **p < 0.01, ***p < 0.001.

Pearson’s correlation was used to calculate the correlation between functional connectivity changes and reaction time before and after sleep deprivation, to explore the relationship between changes in functional connectivity and PVT, as shown in Figure 7. An increase in reaction time was significantly positively correlated with increased functional connectivity within the SN [ACC-Alnsula(R), SN] (*r* = 0.54, *p* < 0.01), and negatively correlated with decreased functional connectivity in the SN and VN [SMG(R)- lateral (R), SN-VN] (*r* = −0.208, *p* = 0.35), but no statistical difference was observed.

## Discussion

In this study, we explored the effects of 36 h of TSD on the functional connectivity of vigilant attention and salience networks in large-scale brain networks. We found that after 36 h of TSD, participants’ vigilance decreased substantially, their responses became more sluggish, and the frequency of wandering increased (Deurveilher et al., 2015). The visual-parietal functional connectivity was decreased, indicating that the brain’s response to visual stimuli processing was reduced, and perceptual processing was impaired, whereas, the anterior cingulate cortex functional connectivity with the insula was enhanced, showing functional compensation, to maintain a certain level of wakefulness. We also found a positive correlation between increased functional connectivity between the ACC and insula and the decline in vigilance attention performance. This suggests that the impairment of vigilance function, in participants with sleep deprivation, may be related to weakened information acquisition, reduced processing ability, and the disruption of functional brain networks (De Havas et al., 2012). However, a compensatory higher cognitive function emerges, suggesting a rebalancing of the brain network.

The ability of the brain to process external information is impaired after sleep deprivation, which is corroborated by our finding of decreased visual-parietal functional connectivity. TSD for 36 h leads to instability of the frontoparietal control network and deactivation of the parietal lobes (Chee et al., 2011), resulting in a lack of attention to specific stimuli and impaired allocation of attentional resources, making weak external stimuli less likely to be noticed. Visual networks are mainly involved in visual representation and processing (Grill-Spector and Malach, 2004), and negative activation of the right lateral lobe allows for restricted external stimulus input. Therefore, the decline in visual-parietal connectivity suggests that sleep deprivation impedes the brain’s cognitive information processing processes, especially for visual stimuli, causing the brain’s neuronal information network to exhibit a more complex chaotic pattern.

We found that the functional connectivity of the ACC and insula appeared to be enhanced during impaired cognitive processing in the brain after 36 h of TSD. The activation of the insula, as the core nodes of the salience network, plays a crucial role in processing visual information, and the increased functional connectivity between the insula and ACC, to a certain extent, compensates for the individual’s persistently impaired level of vigilance attention. Therefore, the increase in this part of functional connectivity suggests that the brain may have functional compensation. Whether the compensated higher or lower cognitive function component has been controversial. Our results show that higher cognitive functions need to be compensated, because we found that the functional connectivity of the ACC to the insula, which represents executive function, was enhanced within the salience network. However, this is contrary to our initial hypothesis of decreased insular connectivity. Few researchers have suggested that sleep deprivation reduces the activation of the SN in attention tasks (Ma et al., 2015). The right insula controls the switching of attentional tasks between the default network and the frontoparietal control network. Dysfunction of the right insula is the main cause of the unstable attentional state after sleep deprivation (Sridharan et al., 2008). In contrast, it has been shown that anterior and ventral activation of the prefrontal lobe, associated with response inhibition tasks, is significantly reduced after sleep deprivation; the right ventral prefrontal lobe and anterior insula are activated more strongly to perform the task after sleep deprivation (Chuah et al., 2006). This may result from differences in the type of task selected for the study and the duration of sleep deprivation; our results support the latter. This suggests a rise in the functional connectivity of the insula with the anterior cingulate cortex for the compensatory higher cognitive functions of sleep deprivation.

We also found a positive correlation between changes in the functional connectivity of the anterior cingulate cortex with the insula and the response time to PVT. This suggests a covariate relationship between brain network functional connectivity changes and behavioral changes. The nervous system cannot directly process physical or chemical energy input from the external world. The input needs to be sensorily encoded and converted into neural impulses that the nervous system can receive to produce a series of behavioral responses. In contrast, fMRI indirectly measure neuronal activity by detecting the BOLD signal, and previous studies have shown a strong correlation between resting-state BOLD data and spontaneously fluctuating local field potential correlation. As Liégeois et al. (2019) showed, that by combining resting-state static functional connectivity and dynamic functional connectivity methods, brain network-based functional connectivity was found to fit and predict the psychological and behavioral characteristics of individuals with considerable explanatory power (Liégeois et al., 2019).

This study is the first to investigate the relationship between changes in insular and vigilance function from large-scale brain networks. Our findings suggest a rebalancing of the brain network, with an equilibrium established at a lower level, since SD impairs the cognitive function of the individual, leading to a continuous decrease in the level of vigilant attention (Borragán et al., 2019; Qi et al., 2021). However, the brain appears to compensate functionally for part of the decrease; this compensatory adaptation can offset insufficient cognitive functions (Drummond et al., 2000). Deantoni also found that functional connectivity between navigation-related brain structures increases during relearning in the extended environment after TSD, which represents the use of compensatory brain resources (Deantoni et al., 2021). Overall, the existence of compensatory neural activity is accurate (Muto et al., 2012). This compensatory mechanism is triggered in individuals who experience SD, thus maintaining another dynamic equilibrium of the brain (Liu et al., 2014).

The limitations of this study are as follows: the participants were all male volunteers who received sleep deprivation in the laboratory. Thus, the effects on the female population were not explored. Further, the ecological validity is low, as the study was done in a lab setting; thus, generalization to daily life should be done with caution. Additionally, individual differences in participants, such as susceptibility to sleep deprivation were not considered, as the susceptibility of the same trait to sleep deprivation varies depending on the participant, as well as the type of daily cycle of sleep. Some participants were of the night type, whereas, some were of the early morning type, which should be considered in future studies. Although this study was equipped with a participant to supervise the other participants’ continuous wakefulness for 36 h, it is inevitable that some of them engaged in strenuous activities, such as playing e-sports games, or fell asleep during this period, thus affecting the overall sleep deprivation. Therefore, future studies could include electrophysiological techniques, to ensure that the participant is asleep. The sample size may also influence changes in functional brain connectivity before and after sleep deprivation; further, appropriate sample expansion could ensure more convincing results. Future studies could also investigate the brain mechanisms underlying impaired vigilance in individuals from graph-theoretic-based analyses, such as functional connectivity density, local coherence, and fractional low-frequency amplitude.

This study found that 36 h of TSD impaired visual vigilance function and decreased functional connectivity of the visual and parietal lobes, which may be related to reduced brain information reception. Simultaneously, there was a compensatory organismal brain network with enhanced functional connectivity between the anterior cingulate cortex, which represents an executive function, and the insula, which needs to be demonstrated in future studies.

## Data availability statement

The raw data supporting the conclusions of this article will be made available by the authors, without undue reservation.

## Ethics statement

The studies involving human participants were reviewed and approved by the Ethics Committee of the Beihang University (Beijing, China). The patients/participants provided their written informed consent to participate in this study.

## Author contributions

WF: formal analysis, methodology, validation, investigation, data curation, writing – original draft, and visualization. CD, JC, LW, and TS: investigation and data curation. ZP: methodology and investigation. MX: conceptualization and methodology. LX: software and resources. YT: conceptualization, methodology, resources, writing – review and editing, supervision, and project administration. YS: conceptualization, methodology, resources, writing – review and editing, supervision, project administration, and funding acquisition. All authors contributed to the article and approved the submitted version.

## Conflict of interest

The authors declare that the research was conducted in the absence of any commercial or financial relationships that could be construed as a potential conflict of interest.

## Publisher’s note

All claims expressed in this article are solely those of the authors and do not necessarily represent those of their affiliated organizations, or those of the publisher, the editors and the reviewers. Any product that may be evaluated in this article, or claim that may be made by its manufacturer, is not guaranteed or endorsed by the publisher.
